# Enhanced wheat productivity in saline soil through the combined application of poultry manure and beneficial microbes

**DOI:** 10.1186/s12870-024-05137-x

**Published:** 2024-05-18

**Authors:** Muhammad Junaid Arshad, Muhammad Imran Khan, Muhammad Hayder Ali, Qammar Farooq, Muhammad Iftikhar Hussain, Mahmoud F. Seleiman, Muhammad Ahsan Asghar

**Affiliations:** 1 Institute of Soil and Environmental Sciences, University of Agriculture, Faisalabad, Pakistan; 2 Department of Isotope Biogeochemistry, Helmholtz- Center for Environmental Research- UFZ, Leipzig, Germany; 3 Department of Plant Biology and Soil Science, Universidad de Vigo, Vigo, Spain; 4 Department of Plant Production, College of Food and Agriculture Sciences, King Saud University, P.O. Box 2460, Riyadh, 11451 Saudi Arabia; 5 Department of Biological Resources, Agricultural Institute, Centre for Agricultural Research, ELKH, Brunzvik St, Martonvásár, 2462 Hungary

**Keywords:** Salinity, Phytotoxicity, Stress alleviation, Bacteria, Wheat, Organic amendments

## Abstract

**Background:**

Soil salinity is one of the major menaces to food security, particularly in dealing with the food demand of the ever-increasing global population. Production of cereal crops such as wheat is severely affected by soil salinity and improper fertilization. The present study aimed to examine the effect of selected microbes and poultry manure (PM) on seedling emergence, physiology, nutrient uptake, and growth of wheat in saline soil. A pot experiment was carried out in research area of Institute of Soil and Environmental Sciences, University of Agriculture, Faisalabad, Pakistan. Saline soil (12 dS m^− 1^ w/w) was developed by spiking using sodium chloride, and used in experiment along with two microbial strains (i.e., *Alcaligenes faecalis* MH-2 and *Achromobacter denitrificans* MH-6) and PM. Finally, wheat seeds (variety Akbar-2019) were sown in amended and unamended soil, and pots were placed following a completely randomized design. The wheat crop was harvested after 140 days of sowing.

**Results:**

The results showed a 10–39% increase (compared to non-saline control) in agronomic, physiological, and nutritive attributes of wheat plants when augmented with PM and microbes. Microbes together with PM significantly enhanced seedling emergence (up to 38%), agronomic (up to 36%), and physiological (up to 33%) in saline soil as compared to their respective unamended control. Moreover, the co-use of microbes and PM also improved soil’s physicochemical attributes and enhanced N (i.e., 21.7%-17.1%), P (i.e., 24.1-29.3%), and K (i.e., 28.7%-25.3%) availability to the plant (roots and shoots, respectively). Similarly, the co-use of amendments also lowered the Na^+^ contents in soil (i.e., up to 62%) as compared to unamended saline control. This is the first study reporting the effects of the co-addition of newly identified salt-tolerant bacterial strains and PM on seedling emergence, physiology, nutrient uptake, and growth of wheat in highly saline soil.

**Conclusion:**

Our findings suggest that co-using a multi-trait bacterial culture and PM could be an appropriate option for sustainable crop production in salt-affected soil.

**Supplementary Information:**

The online version contains supplementary material available at 10.1186/s12870-024-05137-x.

## Introduction

The fast-growing world population and increasing food demand require sustainable and resilient agricultural strategies to ensure higher food production. Global climate change and its biotic and abiotic impacts cause continuous decrease in arable lands [1]. Globally, around one-third of the irrigated land has been affected by salinity/sodicity, and it is increasing at a pace of around 2 million hectares (mha) per year [2, 3]. Soil salinity can develop through natural processes or human interventions. Weathering of rock minerals, marine deposition, improper use of organic/inorganic fertilizers, brackish underground water, poor drainage conditions, and soil erosion cause the deterioration of soil that leading to soil salinity [4]. The improper use of fertilizers and brackish underground water are also the main sources of salt accumulation in soil that pose detrimental effects on current food supplies [5, 6]. In addition, the geological and chemical properties of soil along with local hydrological and climatic factors, are associated with soil salinization [7].

Salinization alters the biological and physicochemical properties of soil and plants by producing specific ions toxicity, osmotic imbalance, and unavailability of essential nutrients [8]. Excessive Na^+^ in the soil competes with potential nutrients (such as K^+^), making them unavailable for plant uptake [9]. Therefore, ever-increasing soil salinity reduces aggregate formation, microbial biomass, water infiltration, and soil aeration that impairs plant physiology by changing osmotic potential and stomatal behaviors [10]. Moreover, lesser adsorption of sodium ions on soil particles causes sodium dominance in the soil solutions leading to soil dispersion, deficiency of divalent cations, and eventually to soil clogging upon drying [11]. It has been reported that salinity could cause a 50% yield reduction in arid and semi-arid regions of the world [6].

Scientists and farmers need to adopt cost-effective and ecologically acceptable techniques such as the incorporation of organic materials including poultry manure (PM), animal dung, farm waste, and crop residues into soils to reduce the deleterious effects of salinity on plants [12, 13]. In fact, by adopting certain practices, the waste organic materials can be altered into useful products [14]. The PM provides a higher amount of macronutrients like nitrogen (∼ 4.5%), phosphorus (∼ 3.5%), and potassium (∼ 3%) and various micro-nutrients for healthy plant growth [15–17]. Moreover, the PM is known to nourish the soil by improving soil porosity, water-holding capacity, carbon stock, nutrient (NPK) cycling, and dissolution of rock minerals [18]. Several studies are reporting the effects of PM on wheat grown in saline soil [19–21], but there is limited research showing the combined application of PM and microbial culture on the growth and yield of wheat plants grown in salt-affected soil.

The use of naturally occurring plant growth-promoting rhizobacteria (PGPRs) in crop production is also trending nowadays for sustainable agriculture [22]. It has been reported that a variety of isolated microbes having growth-promoting characteristics from genera *Microbacterium*, *Acinetobacter*, *Actinobacteria, Azotobacter*, and *Pseudomonas* are effective in alleviating biotic and abiotic stresses in plants [23–25]. Microbes could improve plant growth and physiological traits by producing ACC-deaminase, siderophores, P-solubilization, and indole-3-acetic acid (IAA) even under abiotic stresses [26, 27]. In return, plants release a massive amount of vitamins, organic acids, sugars, amino acids, and related compounds to attract a diversified microbial population near the root zone [28, 29]. These microbes improve the nutritional status of soil along with soil reclamation. Moreover, microbes along with an appropriate organic material such as PM have the potential to reclaim saline soil by improving the biological and physicochemical properties of soil [30]. Hence, it is essential to enrich rhizospheric soil with beneficial microbes and organic waste like PM for sustainable agriculture [31].

Wheat (*Triticum aestivum* L.), a staple food and a cheaper source of protein, fiber, carbohydrates, minerals, vitamins, and gluten, contributes around 35% of the world’s food grains [32, 33]. In 2020, the world’s wheat production was around 760 million tonnes (Mt) [34]; however, according to FAO, wheat production should be raised to 850 Mt by the year 2050 to overcome the food demand of the global population [35]. To fulfill the world’s food demand, cereal crops such as wheat should be grown on marginal lands and problematic (i.e., saline/sodic) soils through nutrient management and suitable soil amendments [36]. The addition of beneficial microbes and organic wastes in soils could be useful in improving soil health and crop yield for sustainable food production [37, 38]. Therefore, the main aim of this study was to assess the potential of two microbial strains i.e., MS1 (*Alcaligenes faecalis* MH-2) and MS2 (*Achromobacter denitrificans* MH-6) alone or in combination with PM in improving the physiology, growth, nutrient contents, and yield attributes of wheat in saline soils for sustainable crop production.

## Materials and methods

### Collection, preparation, and pre-analysis of the soil

The soil was collected from the agronomic field of the Institute of Soil and Environmental Sciences (ISES), University of Agriculture, Faisalabad (UAF). Soil was collected from a depth of 0–30 cm with the help of a soil sampling auger. Firstly, the soil was cleaned for pebbles and crop residues, then air-dried and sieved by passing through a 2 mm sieve. For the pre-analysis of soil, water and PM, a series of laboratory experiments were performed to determine the various physicochemical attributes following standard protocols as described in our previous studies [13, 27, 29]. Results of analyses are provided in Table 1. To prepare saline soil (by spiking) having electrical conductivity (EC) of 12 dS m^− 1^, a measured amount of sodium chloride (99.9% pure analytical grade) was added to respective pots [13]. However, the threshold limit of salinity for wheat crop is 6 dS m^− 1^. After that, the soil was kept in the shade for 20 days to homogenize the added material in the soil [39].

**Table 1:** Physicochemical attributes of soil, water and poultry manure used in this study

Properties	Water		Soil		Poultry manure	
	Value	Unit	Value	Unit	Value	Unit
Sand	-	-	55 ± 3	(%)	-	
Silt	-	-	24 ± 1	(%)	-	
Clay	-	-	21 ± 1	(%)	-	
Saturation percentage	-	-	21.5 ± 1.10	(%)	-	
pH	7.40 ± 0.02	-	7.30 ± 0.08	-	6.24 ± 0.02	
EC	0.78 ± 0.03	dS m^-1^	1.22 ± 0.04	dS m^-1^	-	
CEC	-	-	14.2 ± 1.12	cmol_c_kg^-1^	-	
Organic matter	-	-	0.49 ± 0.02	(%)	22.4 ± 0.60	(%)
Total N	-	-	0.19 ± 0.01	(%)	2.08 ± 0.12	(%)
Available P	-	-	0.04 ± 0.01	(%)	1.48 ± 0.04	(%)
Soluble K^+^	-	-	0.06 ± 0.01	(%)	1.56 ± 0.06	(%)
Soluble Na^+^	0.81 ± 0.04	mmolc L^-1^	48.8 ± 0.14	mmol_c_kg^-1^	-	
Ca^2+^ + Mg^2+^	6.99 ± 0.17	mmolc L^-1^	14.8 ± 0.32	mmolc/L	-	
Soluble carbonates	1.33 ± 0.07	mmolc L^-1^	3.24 ± 0.06	meq L^-1^	-	
Soluble bicarbonates	2.74 ± 0.12	mmolc L^-1^	13.5 ± 0.25	meq L^-1^	-	
Soluble chloride	2.61 ± 0.19	mmolc L^-1^	9.97 ± 0.63	meq L^-1^	-	

EC: Electrical conductivity; CEC: Cation exchange capacity; Na^+^: Sodium; K^+^: Potassium; P: Phosphorus; N: Nitrogen; Ca^2+^ + Mg^2+^: Calcium and magnesium

### Preparation of microbial culture

The tryptic soy broth (TSB) medium was prepared in the Soil and Environmental Microbiology Lab in ISES, UAF. The medium was autoclaved twice before inoculation and the pH of the medium was maintained at 7.0 ± 0.2. Two microbial strains that were previously isolated from the hydrocarbons-contaminated sites and identified as *Alcaligenes faecalis* MH-2 with accession number ON7114529 (MS1) and *Achromobacter denitrificans* MH-6 with accession number ON7114531 (MS2), were inoculated in TSB broth and incubated at 25 ± 3 ˚C at 150 rpm for 96 h [27]. After the incubation, the microbial growth was estimated by measuring the optical density (OD_600_) of the cultures [29] and then the cultures were further used in the pot experiment.

### Experimental setup

A pot experiment was carried out in the experimental area of the ISES, UAF, Pakistan (31°25′59.7″N 73°04′20.1″E) to evaluate the effects of selected microbial strains and PM in saline and non-saline soil. Wheat seeds (variety, Akbar-2019) were collected from Ayyub Agricultural Research Institute (AARI), Faisalabad, Pakistan. This variety was selected due to its higher yield potential and rust-resistance capabilities. The PM was obtained from the farm area of the Department of Agronomy, UAF.

For this experiment, each plastic pot having the dimension of L (33) × W (25) × D (25) was filled with 10 kg of soil. The PM (@ 2%) and 50 mL of microbial culture (having approximately OD_600_ value of 0.5) were added to the selected pots [40]. The recommended amounts of N, P, and K fertilizers i.e., 120 kg N ha^− 1^ as urea, 90 kg P ha^− 1^ as single super-phosphate, and 60 kg K ha^− 1^ as potassium sulfate, respectively were applied to all pots [41, 42]. All fertilizers were applied before seed sowing; however, urea was applied in three splits. Seven wheat seeds were sown in each pot (at a field capacity of 75%) and a total of sixteen treatments in triplicates (please see the treatment details in Table S1) were prepared and placed by following a completely randomized design (CRD). The climatic information during the experiment was recorded and is provided in Table 2. The crop was harvested after 140 days of sowing and the plant’s growth, physiological, and yield attributes were noted during or after experiment by following the standards protocols.

**Table 2:** Average meteorological data collected during the experimental period

Months	Sunshine (Hours)	Temperature (°C)	Rainfall (mm)	Humidity (%)	Evaporation rate (mL)
November	6–8	17.5–30.0	0.00	48–86	0.7–3.8
December	3–9	10.5–18.5	0.40	69–94	0.2–2.8
January	2–9	9.5–14.5	48.4	74–99	0.3–1.4
February	4–10	13.5–19.5	10.2	73–96	0.4-4.0
March	6–11	18.5–29.0	2.10	48–83	1.5–7.6

### Seedling emergence and physiological attributes of wheat plants

After sowing, the seedling emergence was observed till the constant count (i.e., 8th day after sowing). After that, thinning was done to maintain a constant count of 3 seedlings per pot. Watering of the plants was done as per requirement. After 45 days of seed sowing, different physiological parameters such as photochemical quantum yield (YII), fluorescence yield (Ft), electron transport rates (ETR) and photosynthetically active radiation were measured by photosynthetic yield analyzer (MINI-PAM-II, WALZ Mess-und Regeltechnik, Germany) [43]. All these parameters were checked on a fully turgid flag leaf and a bright sunny day at 12:00–2:00 p.m. The SPAD value from the flag leaf was also measured by a SPAD meter (SPAD-502, Konica Minolta, Europe) after 45 days of seed sowing [44].

For the chlorophyll pigment analyses, 0.20 g fresh samples of plants were obtained from the newly matured leaves. This sample was added to methanol and crushed in a mortar with the help of a pestle. After that, these samples were vortexed at 4000 rpm for 30 min to deposit the debris. The supernatant was used further to estimate the carotenoids, Chl a, Chl b, and Total Chl contents of all samples by running them on a UV-visible spectrophotometer with various wavelengths. Equations 1–4 are used to calculate the extent of pigmentation in the leaves of wheat plants.

1 $${\rm{Carotenoids = V/1000 \times W \times }}\left[ {{\rm{4}}{\rm{.16 }}\left( {{\rm{O}}{{\rm{D}}_{{\rm{480}}}}} \right){\rm{ -- 0}}{\rm{.89 }}\left( {{\rm{O}}{{\rm{D}}_{{\rm{663}}}}} \right)} \right]$$

2 $${\rm{Chl}}\,{\rm{a}}\,{\rm{ = }}\,{\rm{V/1000}}\,{\rm{ \times }}\,{\rm{W \times }}\,\left[ {{\rm{12}}{\rm{.7}}\,\left( {{\rm{O}}{{\rm{D}}_{{\rm{663}}}}} \right)\, - {\rm{2}}{\rm{.69}}\,\left( {{\rm{O}}{{\rm{D}}_{{\rm{645}}}}} \right)} \right]$$

3 $${\rm{Chl b = V/1000 \times W \times }}\left[ {{\rm{22}}{\rm{.9 }}\left( {{\rm{O}}{{\rm{D}}_{{\rm{645}}}}} \right){\rm{ -- 4}}{\rm{.68 }}\left( {{\rm{O}}{{\rm{D}}_{{\rm{663}}}}} \right)} \right]$$

4 $${\rm{Total Chl = V/1000 \times W \times }}\left[ {{\rm{20}}{\rm{.2 }}\left( {{\rm{O}}{{\rm{D}}_{{\rm{645}}}}} \right){\rm{ + 8}}{\rm{.02 }}\left( {{\rm{O}}{{\rm{D}}_{{\rm{663}}}}} \right)} \right]$$

W, Fresh leaf weight (mg); V, Methanol volume used in extract (ml).

### Membrane stability index and relative water contents of plants

Fully expanded flag leaves were collected from each replication. A 0.20 g fresh leaves were added in each test tube containing 10 mL of distilled water. One set of test tubes was placed in the water bath at 40 ^o^C for 30 min to obtain EC_1_. For EC_2_, the plant samples were kept in the same water bath for 10 min at 100 ^o^C. By using Eq. 5, the membrane stability index was calculated (Sairam et al., 2002).

5 $${\rm{MSI = }}\left[ {{\rm{1}} - \left( {{\rm{EC1/EC2}}} \right)} \right]{\rm{ \times 100}}$$

Similarly, the relative water contents of fresh flag leaves were determined by following the protocols of Ali et al. [27]. Briefly, 0.5 g fresh leaves samples were soaked in a test tube containing distilled water. After 4 h, the leaves were carefully pulled out from test tubes to gain fully turgid leaves weight. Thereafter, these samples were kept in an oven at 65 °C (until a constant weight was observed) to determine the dry weight. Relative water loss from leaves was calculated by the following equation (Eq. 6).

6 $${\rm{RWC = }}\left[ {\left( {{\rm{FW}} - {\rm{DW}}} \right){\rm{/}}\left( {{\rm{TW}} - {\rm{DW}}} \right)} \right]$$

Where FW is fresh weight, TW is turgid weight, and DW is dry weight.

### Growth and yield attributes of wheat plants

Wheat crop was harvested after 140 days of sowing and the plant’s growth and yield attributes were noted by following the standards protocols. Briefly, the plant length (including root, shoot, and spike) was measured at the time of harvesting by carefully uprooting the plants and measuring the length with a measuring tape. Similarly, the plant fresh biomass was obtained by placing the samples on a portable weight balance right after the harvest. However, the dried biomass was obtained by placing the fresh samples in a shade for 2 days and then placing them in an oven for 65 °C till the obtainment of constant value. Moreover, the number of tillers and spikes from each plant was counted and an average of three plants from each pot was noted. Similarly, the spikelets and number of grains per spike were also counted manually on the same day.

### Chemical analysis of wheat plants

Nitrogen (N), phosphorous (P), potassium (K), and sodium (Na+) were measured by following standard procedures. Total N was assessed by the Kjeldahl digestion method as described by Davidson et al. [45] using the Kjeldahl apparatus (DF-4 S Mitamura Riken Kogyo Inc. Japan). For the estimation of P, K, and Na^+^, the wet digestion method was used as explained by Estefanet al. [46]. The digestion of the oven-dried samples was done with a di-acid (HNO_3_; HClO_4_ ratio of 2:1) mixture. One gram of dried plant sample was kept overnight in a 10 mL di-acid mixture. The material was digested the next day with the help of a hot plate. After cooling, distilled water was added to the material to make up a final volume of 50 mL. By using Whatman’s filter No. 42, digested liquid was filtered and kept air-tight at room temperature before analysis. Furthermore, P in digested samples was measured through a UV-visible spectrophotometer at 430 nm wavelength. Na^+^ and K were determined by a flame photometer (FP7, Jenway, Essex, UK) as illustrated by Banerjee and Prasad [47].

### Statistical analysis

The data obtained from this experiment were statistically analyzed using three-factor factorial CRD (completely randomized design) by the computer-based software STATISTIX 8.1. The variance between treatment means was calculated using Tukey’s honest significant difference (HSD) test [48].

## Results

### Seedling emergence and physiological attributes of wheat plants

Findings from the present study indicated that soil salinization substantially reduced seedling emergence (Table 3). Irrespective of treatments, seeds started to emerge after three days of sowing in most of the pots with only a few exceptions. The individual and co-addition of MS1 (i.e., *Alcaligenes faecalis* MH-2), MS2 *(Achromobacter denitrificans* MH-6), and PM demonstrated a marginal to substantial increase in seedling emergence when compared with a respective control treatment (Table 3). However, the saline control showed 23.5% lesser seedling emergence when compared with non-saline control. The application of MS1, MS2, MC, and PM in saline soil showed higher seedling emergence over respective saline control. Moreover, the co-addition of MS2 and PM along with MC and PM showed (31.6–38.0%) more seedling emergence when compared with un-inoculated and unamended saline control, reflecting the important role of microbes and PM addition in alleviating the salt stress in soil (Table 3).

**Table 3:** Effects of beneficial microbes and poultry manure on seed germination and physiological attributes of wheat plants grown in saline soil

Treatments	SG (%)	SPAD value	Ft (µmolm^2^s^–1^)	PAR (µmolm^2^s^–1^)	YII (µmolm^2^s^–1^)	ETR (µmolm^2^s^–1^)	MSI (%)	RWC (%)
Control	80.9 ± 4.76a**–**c	39.7 ± 0.97 h**–**j	428 ± 22.7f**–**h	1160 ± 20.1 h**–**j	0.43 ± 0.01df	399 ± 20.0 g**–**i	65.6 ± 1.56f**–**i	68.5 ± 4.52b**–**e
P + MS1	85.7 ± 8.26a**–**c	47.2 ± 1.70d**–**g	559 ± 17.4c**–**e	1283 ± 11.0e**–**g	0.46 ± 0.01c**–**e	469 ± 10.1d**–**g	67.9 ± 2.04e**–**h	71.9 ± 3.99b**–**e
P + MS2	90.4 ± 4.77ab	47.9 ± 1.85c**–**f	608 ± 11.6b**–**d	1339 ± 17.2de	0.47 ± 0.01c**–**e	510 ± 21.7c**–**e	69.7 ± 1.46d**–**g	73.8 ± 5.23a**–**e
P + MC	95.2 ± 4.77a	53.2 ± 2.56c**–**e	628 ± 11.1a**–**d	1386 ± 5.81 cd	0.48 ± 0.01 cd	529 ± 11.8b**–**e	71.7 ± 1.89c**–**f	75.8 ± 3.09a**–**e
P + PM	95.2 ± 4.77a	54.3 ± 1.15b**–**d	656 ± 39.5a**–**c	1419 ± 9.91 cd	0.50 ± 0.02bc	558 ± 13.0b**–**d	76.5 ± 2.07a**–**e	77.9 ± 2.36a**–**d
P + MS1 + PM	95.2 ± 4.77a	56.2 ± 1.28a**–**c	663 ± 11.6ab	1460 ± 12.3bc	0.51 ± 0.01bc	573 ± 19.6a**–**c	78.8 ± 1.44a**–**c	80.8 ± 4.46a**–**c
P + MS2 + PM	100 ± 0.00a	57.9 ± 1.57ab	686 ± 30.1ab	1525 ± 20.4b	0.56 ± 0.01ab	618 ± 14.4ab	80.4 ± 0.91ab	84.1 ± 2.28ab
P + MC + PM	100 ± 0.00a	59.7 ± 2.31a	727 ± 30.7a	1651 ± 11.1a	0.60 ± 0.01a	656 ± 14.6a	82.3 ± 1.94a	90.8 ± 1.88a
P + S	61.9 ± 4.77c	34.1 ± 2.59j	335 ± 14.2 h	972 ± 4.98k	0.36 ± 0.01 g	323 ± 17.8i	58.1 ± 1.87i	58.2 ± 2.23e
P + S + MS1	66.6 ± 4.77bc	38.0 ± 1.72ij	418 ± 15.6gh	1028 ± 40.3k	0.38 ± 0.00 fg	368 ± 34.5hi	59.3 ± 2.10i	60.3 ± 0.84de
P + S + MS2	66.6 ± 4.77bc	40.4 ± 0.73 g**–**j	435 ± 15.8f**–**h	1070 ± 44.2jk	0.39 ± 0.01 fg	399 ± 18.6 g**–**i	60.5 ± 1.83hi	61.6 ± 4.18de
P + S + MC	80.9 ± 4.77a**–**c	43.2 ± 1.70f**–**i	470 ± 14.4e**–**g	1131 ± 5.45ij	0.39 ± 0.01 fg	414 ± 12.0f**–**i	62.8 ± 1.01 g**–**i	62.5 ± 3.24de
P + S + PM	80.9 ± 4.77a**–**c	45.5 ± 1.39e**–**h	475 ± 4.36e**–**g	1152 ± 5.54 h**–**j	0.41 ± 0.01e**–**g	436 ± 9.01e**–**h	65.5 ± 1.69f**–**i	64.9 ± 2.81c**–**e
P + S + MS1 + PM	85.7 ± 8.26a**–**c	47.4 ± 1.49c**–**g	489 ± 6.66e**–**g	1194 ± 11.3 g**–**i	0.41 ± 0.01e**–**g	444 ± 10.4e**–**h	69.2 ± 0.69e**–**g	65.7 ± 3.75c**–**e
P + S + MS2 + PM	90.5 ± 4.77ab	50.8 ± 1.95b**–**e	502 ± 18.0e**–**g	1232 ± 4.41f**–**h	0.44 ± 0.01d**–**f	479 ± 20.2c**–**g	72.9 ± 1.39b**–**f	69.4 ± 3.39b**–**e
P + S + MC + PM	100 ± 0.00a	54.4 ± 0.93a**–**c	527 ± 18.6d**–**f	1329 ± 14.4d**–**f	0.49 ± 0.01 cd	509 ± 26.1c**–**f	77.8 ± 1.01a**–**d	73.8 ± 2.63a**–**e

Means and standard errors of triplicate values of each treatment are presented. Tukey’s test (*P* ≤ 0.05) shows that the means with different letters are significantly different from each other. P, Plant; MS1, Microbial strain 1 (i.e., *Alcaligenes faecalis* MH-2); MS2, Microbial strain 2 (i.e., *Achromobacter denitrificans* MH-6); MC, Microbial consortium (i.e., *Alcaligenes faecalis* MH-2 and *Achromobacter denitrificans* MH-6); PM, Poultry manure; S, Salinity; SG, Seed germination; Ft, Fluorescence yield; PAR, Photosynthetically active radiation; YII, Effective PSII quantum yield; ETR, Electron transport rate; MSI, Membrane stability index; RWC, Relative water content

**Fig. 1:**
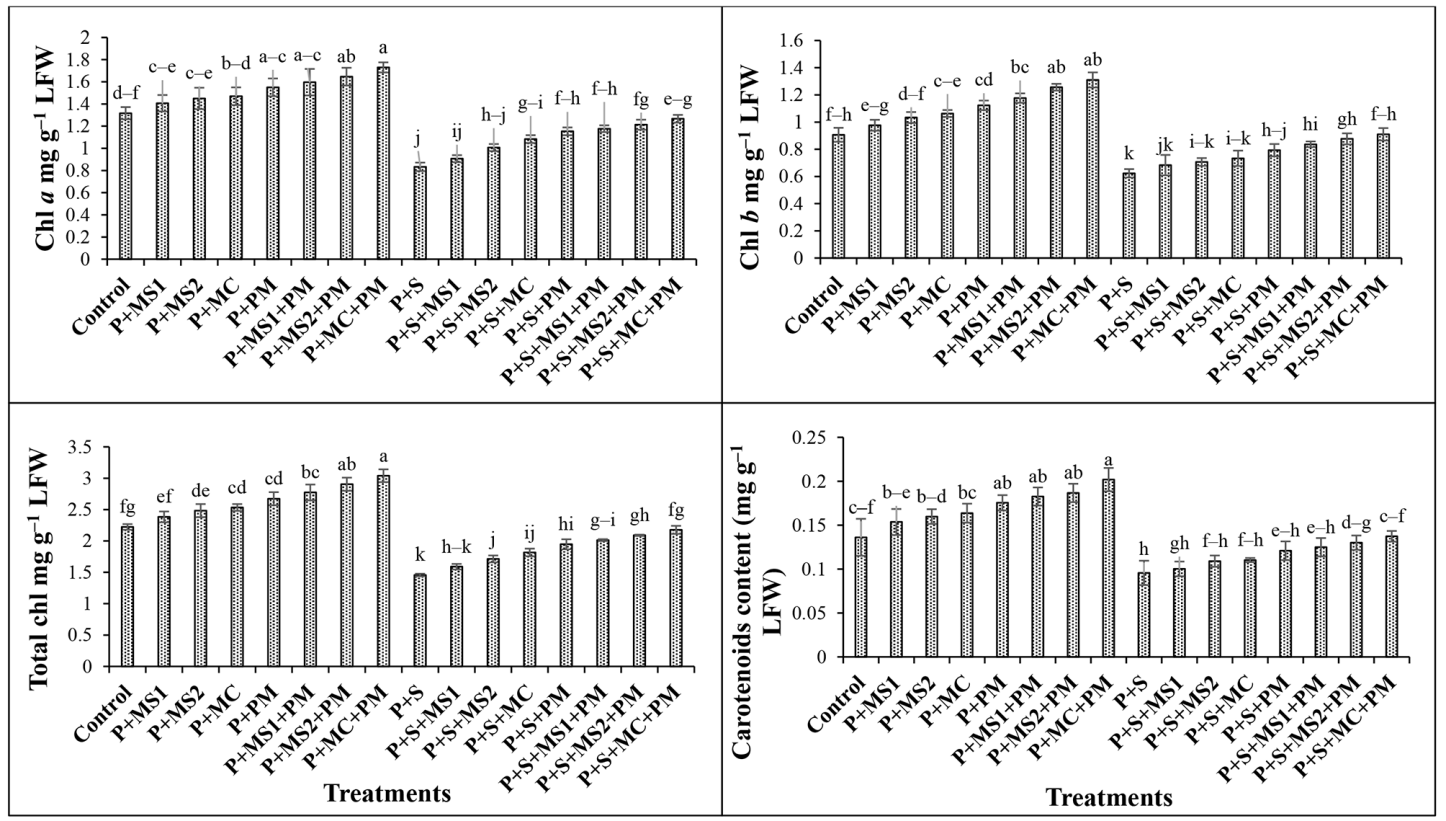
Effects of beneficial microbes and poultry manure on photosynthetic pigments of wheat plants grown in saline soil. Columns and error bars represent the means and standard errors of triplicate values of each treatment, respectively. Tukey’s test at *P* ≤ 0.05 shows that the means with different letters are significantly different from each other. P, Plant; MS1, Microbial strain 1 (i.e., *Alcaligenes faecalis* MH-2); MS2, Microbial strain 2 (i.e., *Achromobacter denitrificans* MH-6); MC, Microbial consortium (i.e., *Alcaligenes faecalis* MH-2 and *Achromobacter denitrificans* MH-6); PM, Poultry manure; S, Salinity; Chl. *a*, Chlorophyll *a*; Chl. *b*, Chlorophyll *b*; Total Chl., Total Chlorophyll; LWF, leaf fresh weight

Furthermore, a significant decrease in physiological attributes of wheat plants was observed as compared to their respective control (Tables 3 and Fig. 1). Around 14.9%, 21.7%, 16.2%, 16.1%, 19.2%, 11.5%, 14.9%, 21.2%, 27.6%, 23.6%, and 29.7% reduction in SPAD value, Ft, PAR, YII, ETR, MSI, RWC, Chl *a*, Chl *b*, total Chl and CC was observed in saline treatments as compared to non-saline control. In saline soil, the addition of MSI, MS2, and MC with PM, significantly improved the physiological traits of the plants by 11.3–31.5%, 16.2–33.3%, and 21.1–36.4% as compared to uninoculated and unamended saline control.

### Growth attributes of wheat plants

The salt contamination caused a considerable reduction (i.e., 10.8–36.5%) in all the agronomic attributes i.e., shoot, root and spike lengths, number of tillers per plant, number of spikes per plant, and number of spikelets per spike (Table 4 and Table 5). Similarly, the fresh and dry weights of shoots and roots, number of grains, grains weight per spike, and 100-grain weight of wheat were also decreased in saline soil as compared to non-saline control. The application of individual strains or MC with PM induced higher shoot length (21.8–28.4%), root length (25.2–31.3%), spike length (19.5–28.3%), number of tillers per plant (27.3–33.3%), number of spikes per plant (30.0–36.4%), and number of spikelets per spike (24.5–28.8%) over their respective saline control. Moreover, the shoot fresh weights (29.8–35.3%), shoot dry weights (31.1–36.5%), root fresh weights (22.5–28.6%), root dry weights (28.9–34.5%), number of grains per spike (34.7–38.2%), grains weight per spike (31.6–35.4%) and 100-grains weights (12.5–17.2%) were increased in amended soil when compared with uninoculated and unamended saline control (Table 4 and Table 5). However, the sole application of microbes and PM also improved the growth and yield attributes of wheat plants in saline stress, but the results were non-significant.

**Table 4:** Effects of beneficial microbes and poultry manure on agronomic attributes of wheat plants grown in saline soil

Treatments	SL (cm)	RL (cm)	SpL (cm)	SFW (g)	SDW (g)	RFW (g)	RDW (g)
Control	63.0 ± 3.46e**–**g	38.8 ± 1.50 g**–**i	9.25 ± 0.52bc	5.67 ± 0.19d**–**f	3.23 ± 0.05e**–**g	1.45 ± 0.02f**–**i	0.49 ± 0.01 g**–**i
P + MS1	70.7 ± 0.33c**–**f	44.4 ± 0.92d**–**f	10.3 ± 0.66a**–**c	6.52 ± 0.54c**–**e	3.91 ± 0.32c**–**e	1.54 ± 0.01 fg	0.54 ± 0.01 fg
P + MS2	73.7 ± 1.76c**–**e	46.7 ± 1.24de	10.5 ± 0.43a**–**c	6.96 ± 0.45b**–**d	4.17 ± 0.27b**–**d	1.59 ± 0.03e**–**g	0.56 ± 0.01e**–**g
P + MC	76.0 ± 2.08b**–**e	48.1 ± 1.29 cd	11.0 ± 0.63a**–**c	7.36 ± 0.34a**–**c	4.41 ± 0.21a**–**c	1.77 ± 0.04de	0.62 ± 0.02de
P + PM	81.3 ± 2.96a**–**d	51.8 ± 1.42bc	11.3 ± 0.55a**–**c	7.47 ± 0.15a**–**c	4.48 ± 0.09a**–**c	1.90 ± 0.03 cd	0.67 ± 0.01 cd
P + MS1 + PM	84.3 ± 2.18a**–**c	53.5 ± 0.46ab	12.0 ± 1.09ab	8.07 ± 0.22ab	4.84 ± 0.13ab	2.03 ± 0.06bc	0.71 ± 0.02bc
P + MS2 + PM	90.3 ± 3.84ab	56.4 ± 0.62ab	12.8 ± 0.83a	8.46 ± 0.08a	5.07 ± 0.05a	2.18 ± 0.03ab	0.76 ± 0.01ab
P + MC + PM	93.3 ± 2.60a	58.2 ± 0.55a	13.2 ± 0.79a	8.72 ± 0.48a	5.23 ± 0.29a	2.25 ± 0.03a	0.79 ± 0.01a
P + S	53.7 ± 3.28 g	29.6 ± 0.93k	8.25 ± 0.52c	3.62 ± 0.06 h	2.13 ± 0.06 h	1.15 ± 0.02k	0.37 ± 0.02j
P + S + MS1	57.0 ± 1.73 fg	32.8 ± 1.02jk	8.83 ± 0.55bc	4.04 ± 0.04gh	2.42 ± 0.02gh	1.25 ± 0.01jk	0.44 ± 0.01ij
P + S + MS2	60.7 ± 2.91e**–**g	35.5 ± 0.44ij	9.08 ± 0.44bc	4.24 ± 0.24f**–**h	2.54 ± 0.14f**–**h	1.29 ± 0.02i**–**k	0.45 ± 0.01hi
P + S + MC	61.3 ± 4.05e**–**g	37.0 ± 0.34 h**–**j	9.33 ± 0.51bc	4.58 ± 0.29f**–**h	2.75 ± 0.17f**–**h	1.33 ± 0.04 h**–**j	0.47 ± 0.02hi
P + S + PM	65.0 ± 3.46e**–**g	38.1 ± 0.89hi	9.91 ± 0.60a**–**c	4.76 ± 0.16f**–**h	2.85 ± 0.09f**–**h	1.41 ± 0.01 g**–**j	0.49 ± 0.01f**–**i
P + S + MS1 + PM	68.7 ± 4.91d**–**g	39.6 ± 1.12f**–**i	10.3 ± 0.80a**–**c	5.16 ± 0.19e**–**g	3.09 ± 0.12e**–**f	1.48 ± 0.01f**–**h	0.52 ± 0.01f**–**h
P + S + MS2 + PM	73.0 ± 1.53c**–**e	41.2 ± 0.66f**–**h	10.9 ± 0.55a**–**c	5.35 ± 0.08e**–**g	3.21 ± 0.05e**–**g	1.55 ± 0.03 fg	0.54 ± 0.01 fg
P + S + MC + PM	75.0 ± 2.64b**–**e	43.1 ± 0.49e**–**g	11.5 ± 0.57a**–**c	5.60 ± 0.22d**–**f	3.36 ± 0.13d**–**f	1.61 ± 0.03ef	0.56 ± 0.01ef

Means and standard errors of triplicate values of each treatment are presented. Tukey’s test (*P* ≤ 0.05) shows that the means with different letters are significantly different from each other. P, Plant; MS1, Microbial strain 1 (i.e., *Alcaligenes faecalis* MH-2); MS2, Microbial strain 2 (i.e., *Achromobacter denitrificans* MH-6); MC, Microbial consortium (i.e., *Alcaligenes faecalis* MH-2 and *Achromobacter denitrificans* MH-6); PM, Poultry manure; S, Salinity; SL, Shoot length; RL, Root length; SpL, spike length; SFW, Shoot fresh weight; SDW, Shoot dry weight; RFW, Root fresh weight; RDW, Root dry weight

**Table 5:** Effects of beneficial microbes and poultry manure on yield attributes of wheat plants grown in saline soil

Treatments	NT	NS	NSp	NGS	GWS (g)	100 GW (g)
Control	3.66 ± 0.33a**–**d	3.33 ± 0.33b**–**e	14.7 ± 0.88b**–**d	35.0 ± 2.31ef	0.99 ± 0.04c**–**g	2.81 ± 0.04 cd
P + MS1	4.33 ± 0.33a**–**d	3.66 ± 0.33a**–**e	15.7 ± 1.45a**–**d	39.7 ± 1.45c**–**e	1.06 ± 0.04b**–**f	3.02 ± 0.02bc
P + MS2	4.66 ± 0.33a**–**c	4.00 ± 0.00a**–**d	17.0 ± 0.58a**–**d	41.3 ± 2.03b**–**e	1.13 ± 0.06b**–**e	3.06 ± 0.02b
P + MC	4.66 ± 0.33a**–**c	4.33 ± 0.33a**–**c	17.7 ± 0.88a**–**d	43.7 ± 1.76a**–**d	1.17 ± 0.05a**–**d	3.12 ± 0.03ab
P + PM	4.66 ± 0.33a**–**c	4.33 ± 0.33a**–**c	19.3 ± 0.88a**–**d	45.3 ± 1.20a**–**c	1.19 ± 0.06a**–**c	3.15 ± 0.02ab
P + MS1 + PM	5.00 ± 0.00ab	4.33 ± 0.33a**–**c	20.3 ± 0.88a**–**c	47.1 ± 1.73a**–**c	1.17 ± 0.03a**–**d	3.12 ± 0.03ab
P + MS2 + PM	5.33 ± 0.33a	4.66 ± 0.33ab	21.0 ± 1.53ab	48.7 ± 2.85ab	1.26 ± 0.02ab	3.16 ± 0.03ab
P + MC + PM	5.33 ± 0.33a	5.00 ± 0.00a	22.3 ± 0.88a	51.3 ± 1.08a	1.34 ± 0.04a	3.24 ± 0.03a
P + S	2.66 ± 0.33d	2.33 ± 0.33e	12.3 ± 1.86d	27.0 ± 2.08f	0.63 ± 0.04j	2.34 ± 0.04i
P + S + MS1	3.00 ± 0.00 cd	2.66 ± 0.33de	13.0 ± 1.53 cd	35.7 ± 0.67de	0.75 ± 0.03ij	2.45 ± 0.02hi
P + S + MS2	3.00 ± 0.53 cd	2.66 ± 0.33de	13.6 ± 0.67b**–**d	36.3 ± 0.88de	0.77 ± 0.03 h**–**j	2.41 ± 0.03 g**–**i
P + S + MC	3.33 ± 0.33b**–**d	3.00 ± 0.00c**–**e	14.0 ± 1.15b**–**d	38.7 ± 0.88c**–**e	0.81 ± 0.04 g**–**j	2.52 ± 0.02f**–**h
P + S + PM	3.66 ± 0.33a**–**d	3.33 ± 0.33b**–**e	14.7 ± 2.33b**–**d	39.7 ± 1.20c**–**e	0.89 ± 0.02f**–**i	2.63 ± 0.03f**–**h
P + S + MS1 + PM	3.66 ± 0.33a**–**d	3.33 ± 0.33b**–**e	16.3 ± 2.19a**–**d	41.3 ± 0.88b**–**e	0.92 ± 0.04f**–**i	2.67 ± 0.03e**–**g
P + S + MS2 + PM	4.00 ± 0.53a**–**d	3.66 ± 0.33a**–**e	16.6 ± 2.33a**–**d	42.3 ± 0.88b**–**e	0.95 ± 0.02e**–**i	2.72 ± 0.02d**–**f
P + S + MC + PM	4.00 ± 0.53a**–**d	3.66 ± 0.33a**–**e	17.3 ± 1.20a**–**d	43.7 ± 0.67a**–**d	0.97 ± 0.03d**–**h	2.84 ± 0.02de

Means and standard errors of triplicate values of each treatment are presented. Tukey’s test (*P* ≤ 0.05) shows that the means with different letters are significantly different from each other. P, Plant; MS1, Microbial strain 1 (i.e., *Alcaligenes faecalis* MH-2); MS2, Microbial strain 2 (i.e., *Achromobacter denitrificans* MH-6); MC, Microbial consortium (i.e., *Alcaligenes faecalis* MH-2 and *Achromobacter denitrificans* MH-6); PM, Poultry manure; S, Salinity; NT, Number of tillers; NS, Number of spikes; NSp, Number of spikelets per spike; NGS, Number of grains per spike; GWS, Grain weight per spike; 100 GW, 100 grains weight

### Nutrient uptake and sodium mitigation

Saline soil reduced the nutrient uptake and accumulation in roots and shoots due to higher salt toxicity and the decrease was about 18.0% and 16.7% for N, 23.9% and 27.8% for P, and 25.1% and 29.7% for K, respectively as compared to non-saline control (Table 6). The application of MS1, MS2, and MC with PM in non-saline soil increased the uptake of N (22.5% and 25.1%), P (29.1% and 22.2%), and K (28.5% and 12.4%) in plants (i.e., roots and shoots), as compared to respective non-saline control. Similarly, the individual and/or combined application of isolated microbes and PM increased the uptake of N (20.6% and 17.1%), P (24.1% and 29.2%), and K (28.7% and 25.2%) in roots and shoots as compared to un-inoculated and unamended saline control (Table 6). Moreover, a non-significant trend was observed in the nutrient attributes of the wheat plant under the sole application of MS1 and MS2.

**Table 6:** Effects of beneficial microbes and poultry manure on nutrient attributes of wheat plants grown in saline soil

Treatment	*N* in root (mg kg^–1^)	*N* in shoot (mg kg^–1^)	*P* in root (mg kg^–1^)	*P* in shoot (mg kg^–1^)	K in root (mg kg^–1^)	K in shoot (mg kg^–1^)
Control	231 ± 4.04e**–**g	149 ± 4.93e**–**g	124 ± 3.00f**–**h	73.3 ± 2.95c**–**e	142 ± 2.96 g**–**i	88.9 ± 3.68b**–**d
P + MS1	247 ± 4.09de	157 ± 4.84d**–**f	131 ± 3.94e**–**g	80.2 ± 4.08a**–**d	157 ± 4.48e**–**g	92.2 ± 3.29a**–**c
P + MS2	245 ± 5.04d**–**f	163 ± 2.73c**–**e	137 ± 4.63d**–**f	83.8 ± 5.46a**–**c	163 ± 273d**–**f	93.5 ± 2.20a**–**c
P + MC	257 ± 2.91 cd	171 ± 3.84b**–**d	148 ± 2.31c**–**e	86.4 ± 4.84a**–**c	171 ± 3.84c**–**e	94.7 ± 1.56a**–**c
P + PM	275 ± 5.49bc	180 ± 3.28bc	155 ± 5.49b**–**d	88.9 ± 4.38ab	180 ± 3.28b**–**d	95.3 ± 3.20a**–**c
P + MS1 + PM	281 ± 4.36ab	187 ± 1.76	158 ± 3.21a**–**c	90.0 ± 2.70ab	187 ± 1.76a**–**c	96.5 ± 0.79ab
P + MS2 + PM	286 ± 4.16ab	189 ± 1.33ab	169 ± 4.36ab	91.9 ± 2.65a	189 ± 1.33ab	97.9 ± 0.70ab
P + MC + PM	298 ± 7.21a	199 ± 3.21ab	175 ± 7.05a	94.2 ± 1.79a	199 ± 3.21a	102 ± 1.40a
P + S	189 ± 2.96i	124 ± 2.64a	94.2 ± 3.77j	52.8 ± 3.16f	107 ± 1.76j	62.5 ± 2.49 g
P + S + MS1	205 ± 4.70hi	127 ± 2.33i	101 ± 3.93ij	61.3 ± 1.45ef	127 ± 2.33i	68.1 ± 2.31 fg
P + S + MS2	203 ± 3.71hi	132 ± 3.28hi	108 ± 3.21 h**–**j	63.6 ± 1.20ef	133 ± 3.28hi	69.7 ± 2.33
P + S + MC	216 ± 4.58gh	137 ± 4.36 g**–**i	110 ± 2.33 h**–**j	64.1 ± 1.62ef	137 ± 4.35hi	73.5 ± 2.21 fg
P + S + PM	224 ± 4.41f**–**h	141 ± 4.91 g**–**i	115 ± 2.40 g**–**i	66.7 ± 1.20d**–**f	141 ± 4.91 g**–**i	75.8 ± 1.88e**–**g
P + S + MS1 + PM	221 ± 3.46gh	142 ± 3.61f**–**i	119 ± 2.40f**–**i	71.3 ± 1.45c**–**e	142 ± 3.61 g**–**i	78.0 ± 1.73ef
P + S + MS2 + PM	226 ± 4.36e**–**h	145 ± 3.05f**–**i	122 ± 2.65f**–**h	72.3 ± 1.76c**–**e	145 ± 3.05gh	78.3 ± 1.76ef
P + S + MC + PM	239 ± 3.18d**–**g	149 ± 4.05e**–**h	124 ± 2.65f**–**h	74.7 ± 1.86b**–**e	150 ± 4.06f**–**h	83.7 ± 1.76ef

Means and standard errors of triplicate values of each treatment are presented. Tukey’s test (*P* ≤ 0.05) shows that the means with different letters are significantly different from each other. P, Plant; MS1, Microbial strain 1 (i.e., *Alcaligenes faecalis* MH-2); MS2, Microbial strain 2 (i.e., *Achromobacter denitrificans* MH-6); MC, Microbial consortium (i.e., *Alcaligenes faecalis* MH-2 and *Achromobacter denitrificans* MH-6); PM, Poultry manure; S, Salinity; N, Nitrogen; P, Phosphorus; K, Potassium

**Fig. 2:**
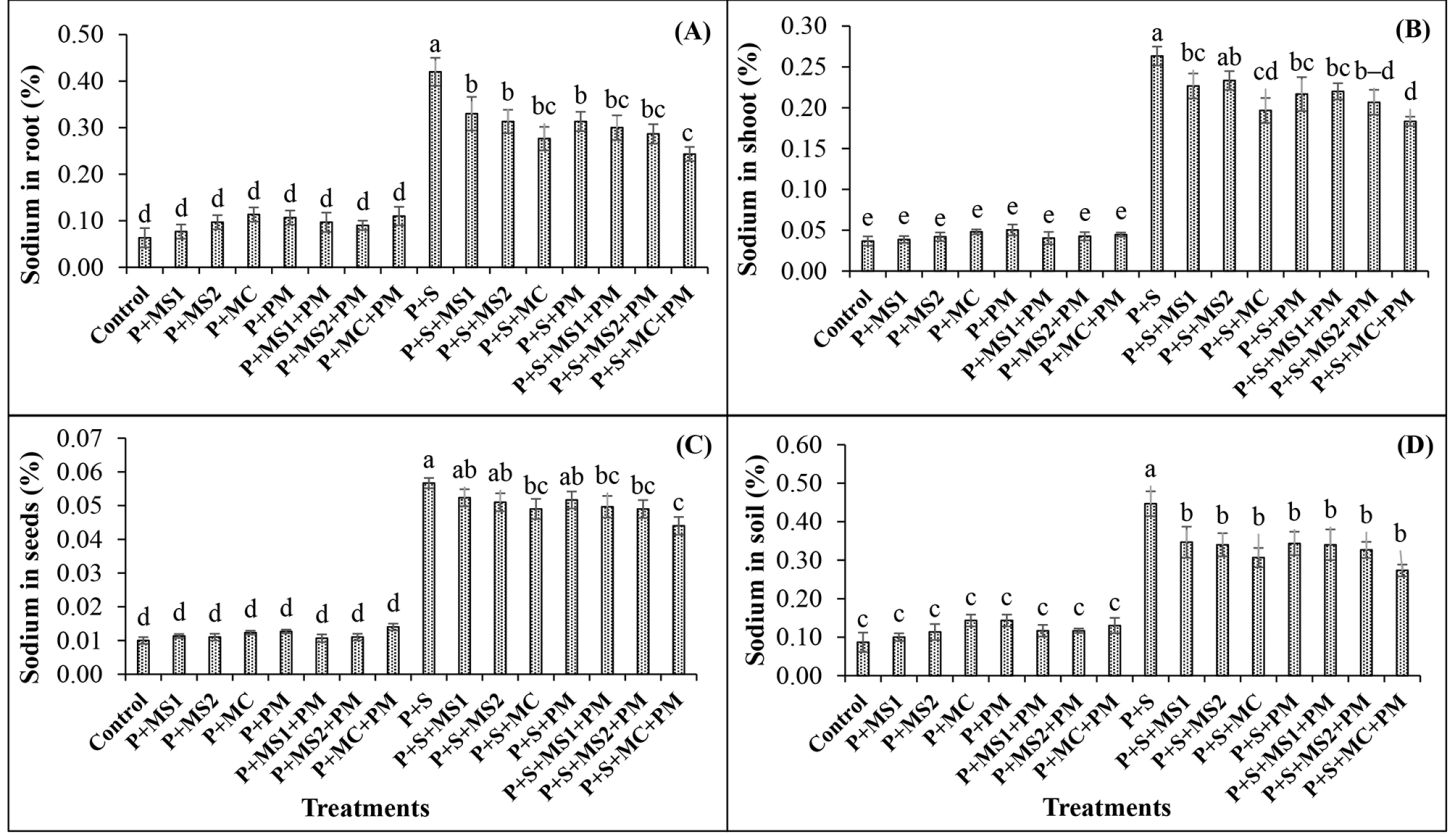
Effects of beneficial microbes and poultry manure on sodium contents of wheat grown in saline soil. Sodium in root (**A**), shoot (**B**), seeds (**C**) and soil (**D**) was analyzed on harvesting (i.e., after 140 days of sowing). Columns and error bars represent the means and standard errors of triplicate values of treatments, respectively. Tukey’s test at *P* ≤ 0.05 shows that the means with different letters are significantly different from each other. P, Plant; MS1, Microbial strain 1 (i.e., *Alcaligenes faecalis* MH-2); MS2, Microbial strain 2 (i.e., *Achromobacter denitrificans* MH-6); MC, Microbial consortium (i.e., *Alcaligenes faecalis* MH-2 and *Achromobacter denitrificans* MH-6); PM, Poultry manure; **S**, Salinity

Maximum concentration of sodium was observed in saline control. The application of bacterial cultures together decreased the sodium contents by around 34.1% in roots, 25.3% in shoots, and 13.5% in seeds than that of saline control (Fig. 2). The application of microbial strains along with PM in saline pots alleviated the salinity stress in the root by 42.1%, in shoots by 30.3%, and in seeds by 22.3% as compared to saline control. Similarly, the co-addition of MC and PM together decreased the sodium contents in the soil by 38.8% when compared with the non-amended saline control (Fig. 2).

### Correlation analysis among wheat attributes

**Fig. 3:**
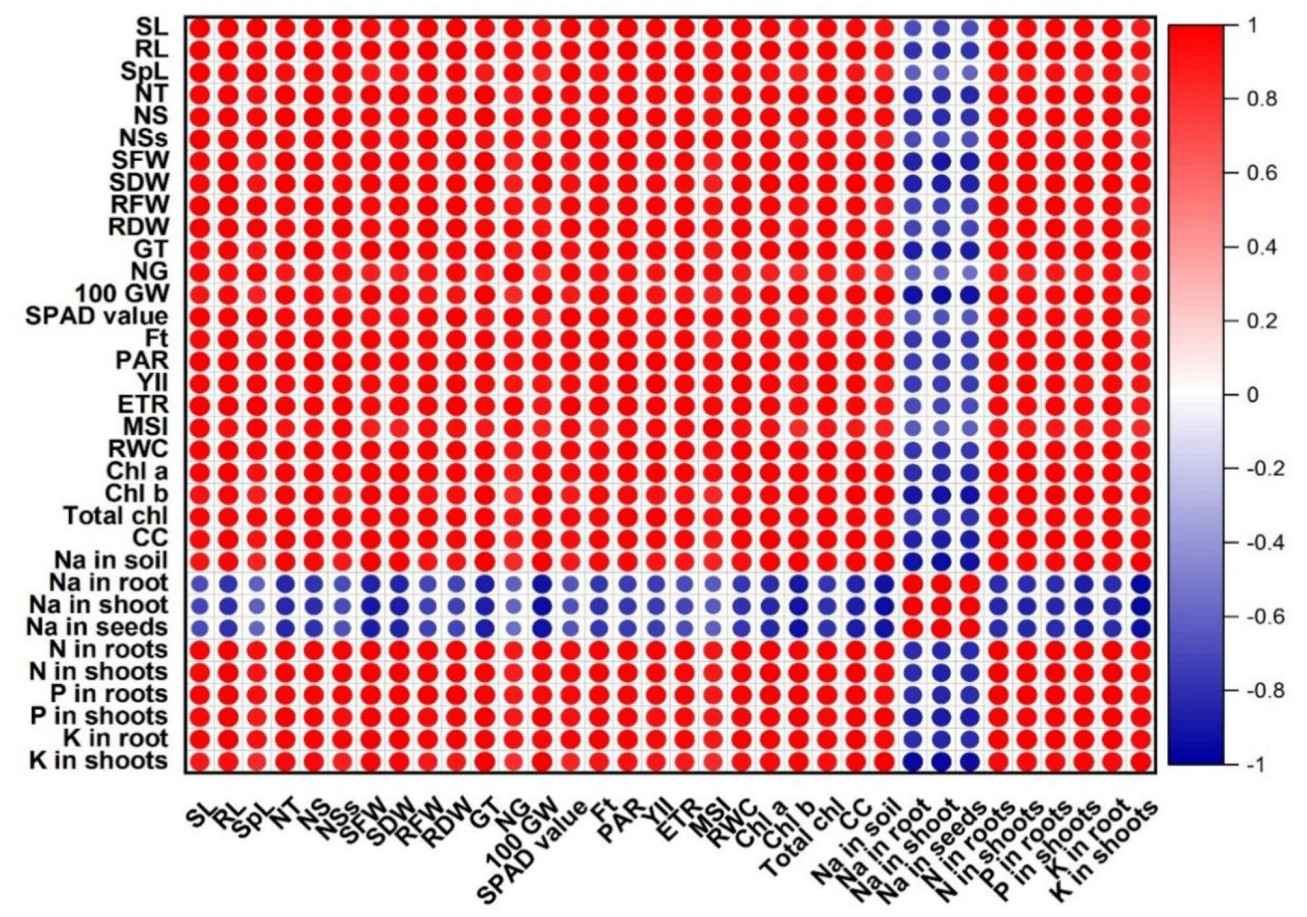
Correlation plot represents a correlation matrix among different growth, physiological, and nutrient attributes of wheat crop grown in saline and non-saline soil. The size of the square shows the strength of the relationship (high, moderate or low) of different attributes of the wheat crop. The dark red and dark blue colors show a highly positive or negative correlation, respectively. The color legend shown on the right side of the correlation plot indicates the corresponding colors and the correlation coefficient. SL, shoot length; RL, root length; SpL, Spike length; NT, No. of tillers per plant; NS, No. of spikes per plant; NSs, No. of spikelets per plant; SFW, shoot fresh weight; SDW, shoot dry weight; RFW, root fresh weight; RDW; root dry weight; GT, Grain weight per spike; NG, No. of grains per spike; 100 GW, 100 Grains weight; SPAD value, Chlorophyll content; Ft, Fluorescence yield; PAR, Photosynthetically active radiation; YII, Quantum yield; ETR, Electron transport rate; MSI, membrane stability index; RWC, relative water contents; Chl *a*, chlorophyll *a*; Chl *b*; chlorophyll *b*; Total Chl, Total chlorophyll; CC, Carotenoids content; Na in soil, Sodium in soil; Na in root, Sodium in roots; Na in shoot, Sodium in shoots; Na in seeds, Sodium in seeds; N in roots, Nitrogen in roots; N in shoots, Nitrogen in shoots; P in roots, Phosphorus in roots; P in shoots, Phosphorus in shoots; K in roots, Potassium in roots; K in shoots, Potassium in shoots

In the present study, a correlation analysis was performed to check the correlation among different growth, physiological, and nutrient attributes of wheat plants in saline and non-saline soil (Fig. 3). The results showed a strong positive correlation among the physiological (e.g., SPAD value, ETR, YII, Ft, and RWC), growth (e.g., shoot and root lengths and dry weights), and nutrient (e.g., N, P, and K content in shoot and roots) attributes of wheat plant when amended with microbes and PM. However, a strongly negative correlation among growth (e.g. root length, number of tillers, number of spikes, and 100-grain weight), physiological (e.g., YII, Ft, and Chl contents), and nutrient (e.g., N, P, and K) traits of wheat plants was seen in saline soil (Fig. 3).

## Discussion

In this study, the physiological, growth, and chemical attributes of wheat plants were reduced in the presence of higher concentrations of salts in the soil. However, the presence of microbes (MS1, MS2, and MC) and PM played an important role in the alleviation of the phytotoxic effects of excessive sodium ions in soil by making them unavailable for plant uptake.

### Effect of microbes and poultry manure on seedling emergence and growth of wheat plants in saline soil

The quality and quantity of the crop yield can be estimated by seedling emergence which directly influences agricultural productivity [49, 50]. In the present study, the saline soil reduced the seedling emergence because of the limited availability of water and nutrients which is required for proper seed imbibition [51, 52]. Moreover, the reduced osmotic potential (by specific ions) disturbs the enzymatic functions [1] and affects the metabolic processes inside the seed that cause embryonic death [53]. The application of microbes and PM reduced the ionic toxicity (i.e., salinity) by stabilizing the soil structure, improving soil drainage, soil porosity and by providing more active sites for ionic sorption [20]. This may also be due to lower soil pH, EC, and sodium adsorption ratio (SAR) that eventually increase the water and nutrient intake in the seeds [54].

Furthermore, in the current study, reduced plant growth was also observed in saline soil. Similar to our study, a significant reduction in roots and shoot lengths and 100-grain weight was also seen by Zhao et al. [55] under salt stress. Similarly, dry and fresh weights were reduced in saline soil and the results are consistent with those of Khan et al. [56]. They also showed a significant reduction (30%) in tillers formation and grain yield of wheat under saline conditions. However, the application of individual microbe and/or their culture along with the PM caused significant impacts on plant growth by triggering nutrient assimilation under salt stress. The mixed culture having ACC deaminase activity may lower the ethylene concentration and reactive oxygen species (ROS) under stress conditions by the production of ACC deaminase and several organic acids [27, 29]. The siderophore activities of the added microbes help to assimilate an adequate amount of iron in plants to strengthen metabolic phenomena like photosynthesis and respiration [57]. Similarly, the P-solubilization characteristics of these microbes enable them to convert insoluble phosphorus to plants available form for proper plant growth [58]. More likely the improved growth in normal or saline soil could be attributed to plant growth-enhancing hormones such as gibberellins and indole-3-acetic acid secreted by the bacteria [29, 59], and this could enhance plants’ resistance against salt stress [60]. Findings of the present work also showed enhanced wheat growth in saline and non-saline soil when PM was applied in combination with microbes. Poultry manure is also a source of carbon (readily available food) which is consumed by microbes to improve their activity and nutrient availability [20, 40]. The PM and microbes play a crucial role in balancing the nutrient ions, increasing microbial activities and improving nutrient availability. It is a fact that microbes and PM increase the availability of macro- and micro-nutrients to plants under the influence of abiotic stresses [61, 62]. Hence, the improved results obtained in this study could be justified by the fact that the use of microbes alone and together with organic amendments improved plant growth under saline stress.

### Effect of microbes and poultry manure on the physiology of wheat plants in saline soil

In this study, the physiological attributes of wheat were also badly affected by salinity. These outcomes support the findings of the latest studies concluding that salt toxicity decreases the chlorophyll contents of plants by affecting chloroplast structure and thus lowering the capability of leaves to capture sunlight for photosynthesis [63, 64]. The impaired physiology might be due to the enhanced level of ROS which causes peroxidation of unsaturated fatty acid, enzyme inhibition, and damages the nucleic acids [65]. Our findings align with the findings of Kareem et al. [66] and Zait et al. [67]. They concluded that salinity alters the biochemical composition of plants and inhibits stomatal conductance. Another reason for impaired physiology is the malfunctioning of stomatal openings, im-proper cell division and elongation that reduces plant growth which might be due to lower water and nutrient intake [68]. Moreover, the values of certain physiological attributes like PAR, YII [69], Ft [70], MSI, and RWC were also adversely affected in saline soil. Our outcomes also endorse the observations of Ahmed et al. [71], indicating the negative effects of salt on the physiological traits of wheat plants.

Co-addition of microbes and PM showed a significant improvement in plant physiology. These findings are consistent with the findings of Ding et al. [21] and Khan et al. [72], who observed improved soil properties and physiological attributes of plants when organic amendments and microbes are applied together. The improved attributes in this work could be due to the formation of complexes in soil (chelation), lowering of pH in the rhizosphere (due to the production of organic acids such as humic and fulvic acids, and amino acids), higher potash solubilization (which regulates the stomatal openings) and more nutrient uptake for proper plant functioning under stressed conditions [73]. The co-use of PM and microbes is beneficial in improving the physiological traits of wheat plants in saline soil.

### Effect of microbes and poultry manure on nutrient uptake by wheat plants in saline soil

The present study also showed the interactive effects of PM and microbes on nutrient assimilation in various plant parts under salt stress. In this study, salt stress significantly reduced the nutrient uptake in plants and the results agree with the findings of Abdul Qadir et al. [74] and Merwad [75] who also showed reduced N, P, and K assimilation in wheat crops grown in saline soil [72, 76]. However, the application of PM alleviated the phytotoxicity of slats and enhanced nutrient uptake. Our results support the findings of Xie et al. [77], who also observed more N, P, and K in different plant tissues when organic amendments were applied to soil. Moreover, PM and microbes release organic substances that precipitate toxic ions from the rhizosphere of soil and alleviate the phytotoxicity of salts. Although the plants uptake fewer nutrients in saline soil, the addition of microbes and PM balances the ionic toxicity and improves the nutrient intake by the production of more root biomass and length [78]. To the best of our knowledge, this is the first study reporting the interactive effects of newly isolated microbes (from hydrocarbon-contaminated sites) and poultry manure on wheat growth under salinity stress.

Findings from the present study enhance our knowledge of plant-microbe interactions in saline soil and provide new information on the usage of microbes and PM together in reducing the impacts of salinity on plant growth and improving crop productivity and this will open new avenues for studying the significance of organic amendments to cope abiotic stresses and improves crop yield.

## Conclusion

This study aimed to alleviate salinity stress and increase wheat plant growth by microbes and organic amendment (i.e., poultry manure). The salinity adversely affected the physiological (up to 30%), agronomic (up to 38%), and biochemical (up to 26%) attributes of wheat plants. However, the co-application of multi-trait bacterial strains and PM together alleviated the negative impacts of salinity on wheat growth and increased the overall efficiency of plants by improving the plant’s physiology (up to 36%), nutrient intake (up to 29%), defense mechanism, and soil biochemical functioning. Furthermore, microbes and PM could precipitate and/or solubilize the nutrients to keep an ionic balance in the nutrition of plants and improve the plant defense mechanism. A quite successful impact of MS2 was seen (as compared to MS1) in terms of promoting plant growth and development and alleviating phytotoxic effects of soil salinity on wheat plants. Findings from this study suggest that the combined use of novel bacterial strains and PM is useful for the alleviation of salinity stress (i.e., up to 62%) in salt-affected soil. However, pot trials under lab conditions are needed to explore the physiological mechanisms behind salt stress mitigation and to investigate the potential of the tested combination of PM and microbes for different crops on salt-affected soil in the field.

### Electronic supplementary material

Below is the link to the electronic supplementary material.

**Supplementary Material 1: Table S1.** Detail of treatments used in this study.

## Data Availability

The original contributions presented in this study are included in the article and/or supplementary material, further inquiries can be directed to the corresponding author.
