# Supplementary material for: Enhanced wheat productivity in saline soil through the combined application of poultry manure and beneficial microbes
Source: BMC Plant Biol. 2024 May 18;24:423. doi: 10.1186/s12870-024-05137-x (PMC11102207; doi:10.1186/s12870-024-05137-x)
Supplement: Supplementary file 1 — Supplementary Material 1: Table S1. Detail of treatments used in this study. [file 12870_2024_5137_MOESM1_ESM.docx]

**Supplementary material**

**Enhanced wheat productivity in saline soil through the combined application of poultry manure and beneficial microbes**

Muhammad Junaid Arshad^1^, Muhammad Imran Khan^1,2,*^, Muhammad Hayder Ali^1^, Qammar Farooq^1^, Muhammad Iftikhar Hussain^3^, Mahmoud F. Seleiman^4^, Muhammad Ahsan Asghar^5^

^1^Institute of Soil and Environmental Sciences, University of Agriculture, Faisalabad, Pakistan

^2^Department of Isotope Biogeochemistry, Helmholtz- Center for Environmental Research- UFZ, Leipzig, Germany

^3^Department of Plant Biology and Soil Science, Universidad de Vigo, Vigo, Spain

^4^Department of Plant Production, College of Food and Agriculture Sciences, King Saud University, P.O. Box 2460, Riyadh 11451, Saudi Arabia

^5^Department of Biological Resources, Agricultural Institute, Centre for Agricultural Research, ELKH, 2 Brunzvik St., 2462 Martonvásár, Hungary

*** Correspondence**: [khanimran1173@yahoo.com](mailto:khanimran1173@yahoo.com)

**Table S1.** Detail of treatments used in this study.

| **Treatments** | **Details** |
| --- | --- |
| Control | Plants grown on non-saline soil |
| P+MS1 | Plants grown on non-saline soil with *Alcaligenes faecalis* MH-2 |
| P+MS2 | Plants grown on non-saline soil with *Achromobacter denitrificans* MH-6 |
| P+MC | Plants grown on non-saline soil with *Alcaligenes faecalis* MH-2 and  *Achromobacter denitrificans* MH-6 |
| P+PM | Plants grown on non-saline soil with poultry manure |
| P+MS1+PM | Plants grown on non-saline soil with *Alcaligenes faecalis* MH-2 and poultry manure |
| P+MS2+PM | Plants grown on non-saline soil with *Achromobacter denitrificans* MH-6 and poultry manure |
| P+MC+PM | Plants grown on non-saline soil with *Alcaligenes faecalis* MH-2, *Achromobacter denitrificans* MH-6, and poultry manure |
| P+S | Plants grown on saline soil |
| P+S+MS1 | Plant grown on saline soil with *Alcaligenes faecalis* MH-2 |
| P+S+MS2 | Plant grown on saline soil with *Achromobacter denitrificans* MH-6 |
| P+S+MC | Plants grown on saline soil with *Alcaligenes faecalis* MH-2 and  *Achromobacter denitrificans* MH-6 |
| P+S+PM | Plants grown on saline soil with poultry manure |
| P+S+MS1+PM | Plants grown on saline soil with *Alcaligenes faecalis* MH-2 and poultry manure |
| P+S+MS2+PM | Plants grown on saline soil with *Achromobacter denitrificans* MH-6 and poultry manure |
| P+S+MC+PM | Plants grown on saline soil with *Alcaligenes faecalis* MH-2, *Achromobacter denitrificans* MH-6, and poultry manure |

P: Plant; MS1, Microbial strain 1 (i.e., *Alcaligenes faecalis* MH-2); MS2, Microbial strain 2 (i.e., *Achromobacter denitrificans* MH-6); MC, Microbial consortium (i.e., *Alcaligenes faecalis* MH-2 and *Achromobacter denitrificans* MH-6,); PM, Poultry manure; S, Salinity.
